# How People Take Cold

**Published:** 1882-02

**Authors:** 


### HOW PEOPLE TAKE COLD.

Not by tumbling into the river and dragging home wet as a drowned rat; not by being pitched into the mud, or spilled out in the snow in sleighing time; not by walking for hours over shoe-top in mud; not by soaking in the rain without an umbrella; not by scrubbing the floor until the un-nameable sticks to you like a wet rag; not by hoeing potatoes until you are in a lather of sweat; not by trying to head a pig in mid-winter, and induce him to run the other way, for he won't do any such thing; not by steaming over the wash-tub; not by essaying to teach Biddy to make mince pies for Christmas, when you don't know how yourself, and then worrying yourself into a perspiration because the pies stuck to the pan, and came out in a muss, forgetting that pie-pans, like people, are rather the better for a little greasing, alias soft soap; these are not the things which give people colds; and yet people are all the time telling us how they "caught their death by exposure." Horace Greeley once said, "O for a leisure week to read books."

*The* TIME *for taking cold, is after your exercise; the* PLACE *is in your own house, or office, or counting-room.*

It is not the act of exercise which gives the cold, but it is the getting cool too quick after exercising. For example, you walk very fast to get to the railroad station, or to the ferry, or to catch an omnibus, or to make time for an appointment; your mind being ahead of you, the body makes an over effort to keep up with it, and when you get to the desired spot, you raise your hat and find yourself in a perspiration; you take a seat, and the forehead will be moist; let the hat remain a few moments and feel the forehead again, and it will be dry, showing that the room is actually cooler than your body, and that with your out-door clothing on, you have cooled off full soon. Among the severest colds I have known men to take, were the result of sitting down to a meal in a cool room, after a walk; or being engaged in writing, have let the fire go out, and their first admonition of it was that creeping chilliness which is the ordinary forerunner of a severe cold. Persons have often lost their lives by writing or reading in a room where there was no fire, although the weather outside was rather uncomfortable. Sleeping in rooms long unused, has destroyed the life of many a visitor and friend. Our splendid parlors, and our nice "spare rooms," help to enrich many a doctor. The cold sepulchral parlors of New York, from May until November, bring disease, not only to visitors, but to the visited; for coming in from domestic occupations, or from the hurry of dressing, the heat of the body is higher than natural, and having no cloak or hat on in going in to meet a visitor, and having in addition but little vitality, in consequence of the very sedentary nature of town life, there is but very little capability of resistance, and a chill and cold is the result.

But *how to cure a cold promptly?* that is a question of life and death to multitudes. There are two methods of universal application: 1st, obtain a bottle of cough mixture, or a lot of cough candy, any kind will do; in a day or two you will *feel* better, and in high spirits; you will be charmed with the promptness of the medicine; make a mule of yourself, by giving your certificate of the valuable remedy, and in due course of time, another certificate will be made for your admission, foot foremost, into "Greenwood."

The other remedy is, consult a respectable resident physician.

### DIGESTION

Is that process which extracts from our food the elements of growth, repair, and sustenance. If the digestion is imperfect, the health of the body becomes imperfect in a few hours; and if by any means digestion ceases altogether, soon after a hearty meal, a man will as certainly die within a few hours, and sometimes almost as suddenly, as if a bullet were shot through his heart. Any great emotion of passion or pleasure, soon after eating, causes death; hence, no highly exciting or momentous news should be communicated, even to the healthiest, let alone the sick and the feeble, immediately after a full repast.

Sometimes the wisest of us will eat too much; for an occasional indiscretion of this kind, two or three teaspoonfuls of strong vinegar afford relief to some persons, but aggravate the evil in a few. The better plan is, to take a long leisure walk in the open air, with a pleasant associate. Keep on walking until entire relief is experienced, and eat no more of anything until next morning, so as to allow the overtaxed stomach to recover its tone, vigor, and elasticity.

If we become conscious of a surfeit after night, and from that or any other cause, a walk is impracticable, a good substitute is found, in standing erect with the clothing removed, except the stockings, mouth closed, and rubbing the region of the stomach, and for a foot around it, with the open hand. Very great relief is often afforded, even in serious cases, within half an hour, by a vigorous manipulation of this sort, taking for breakfast, next morning, a cup of some kind of hot drink and a single piece of dry bread; and for dinner, a bowl of soup with bread crust, and nothing else for that day. The stomach should always be allowed extra rest after overwork.

